# Circularly Polarized Long‐Persistent and Photostimulated Luminescence Enabled through Förster Resonance Energy Transfer and Upconversion Strategies

**DOI:** 10.1002/advs.202523415

**Published:** 2026-01-12

**Authors:** Ruttapol Malatong, Rengo Yoshioka, Dmitry Kovalevskiy, Kentaro Takaji, Hajime Shigemitsu, Kaede Kawaguchi, Yemineni S. L. V. Narayana, Toshiyuki Kida, Ryota Kabe

**Affiliations:** ^1^ Organic Optoelectronics Unit Okinawa Institute of Science and Technology Graduate University (OIST) Onna‐son Okinawa Japan; ^2^ Department of Applied Chemistry Graduate School of Engineering The University of Osaka Suita Osaka Japan; ^3^ Department of Chemistry School of Applied Science and Humanities Vignan's Foundation for Science Technology and Research Andhra Pradesh India

**Keywords:** chiral emitter, circularly polarized long‐persistent luminescence, circularly polarized luminescence, circularly polarized photostimulated luminescence, Förster resonance energy transfer

## Abstract

Circularly polarized luminescence (CPL) has attracted significant attention for applications in displays, data encryption, anti‐counterfeiting, and bioimaging. However, extending the emission lifetime beyond the second timescale remains a challenge. Here, we report circularly polarized long‐persistent luminescence (CP‐LPL) and the first evidence of circularly polarized photostimulated luminescence (CP‐PSL) in purely organic systems. Using the chiral emitter *R/S*‐OBN‐Cz, we establish two complementary design strategies: (i) a three‐component Förster resonance energy transfer (FRET) system, where the energy of long‐lived charge‐separated states between the donor and the acceptor is transferred to the chiral dopant, and (ii) a two‐component upconversion system, where the locally excited state of chiral emitter is restored upon charge recombination. Both approaches result in CP‐LPL with mirror‐image CPL signals. Moreover, in the three‐component FRET system, trapped charges in the chiral dopant can be released upon near‐infrared stimulation, regenerating circularly polarized emission. This work establishes new proof of concept in chiroptical materials research, paving the way toward the practical applications in encrypted optical storage and advanced photonic devices.

## Graphical abstract

Using chiral emitters with circularly polarized luminescence (CPL) characteristics integrated into organic long‐persistent luminescence (OLPL) system demonstrates circularly polarized long‐persistent luminescence (CP‐LPL). Importantly, the Förster resonance energy transfer (FRET)‐based design reveals circularly polarized photostimulated luminescence (CP‐PSL) upon near‐infrared stimulation for the first time.

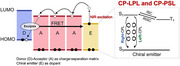

## 1 Introduction

Circularly polarized luminescence (CPL), which involves the emission of right‐ or left‐handed polarized light from chiral luminescent materials, has garnered growing interest due to its potential applications in technologies such as 3D displays, optical data encryption, anti‐counterfeiting, and biomedical imaging [1, 2, 3, 4, 5, 6, 7, 8]. By prolonging the afterglow duration, it becomes easier to distinguish it from the excitation light, enabling the development of applications that differ from those of steady‐state CPL. Considerable efforts have been devoted to long‐lived CPL based on room‐temperature phosphorescence (RTP) and thermally activated delayed fluorescence (TADF) that utilize triplet excitons through intersystem crossing (ISC) [9, 10, 11, 12, 13, 14, 15, 16, 17, 18, 19, 20]. While these approaches enabled lifetimes on the order of seconds, further extension has remained difficult due to the presence of competing nonradiative deactivation of the excited states.

Organic long‐persistent luminescence (OLPL) has recently emerged as a powerful strategy to address this limitation. OLPL systems, a blend of donor and acceptor molecules, upon excitation can generate long‐lived charge‐separated (CS) states, which enable afterglow lasting for hours [21, 22, 23, 24, 25, 26, 27, 28, 29]. To extend CPL far beyond the timescale of conventional RTP or TADF, circularly polarized long‐persistent luminescent (CP‐LPL) system was explored. Previous CP‐LPL study has relied on charge‐transfer (CT) emission from exciplex between a chiral molecule and an achiral host [30]. However, such system typically yields weak CT luminescence, limiting intensity and polarization fidelity.

Beyond persistent luminescence, some OLPL systems were designed to exhibit photostimulated luminescence (PSL), in which stored charges are released upon secondary near‐infrared (NIR) excitation to regenerate luminescence [31]. Achieving circularly polarized PSL (CP‐PSL) would open a new direction in chiroptical materials research by combining persistent, re‐stimulable luminescence with circular polarization, enabling unique functionalities for data storage and secure photonic applications [32, 33, 34].

**FIGURE 1:**
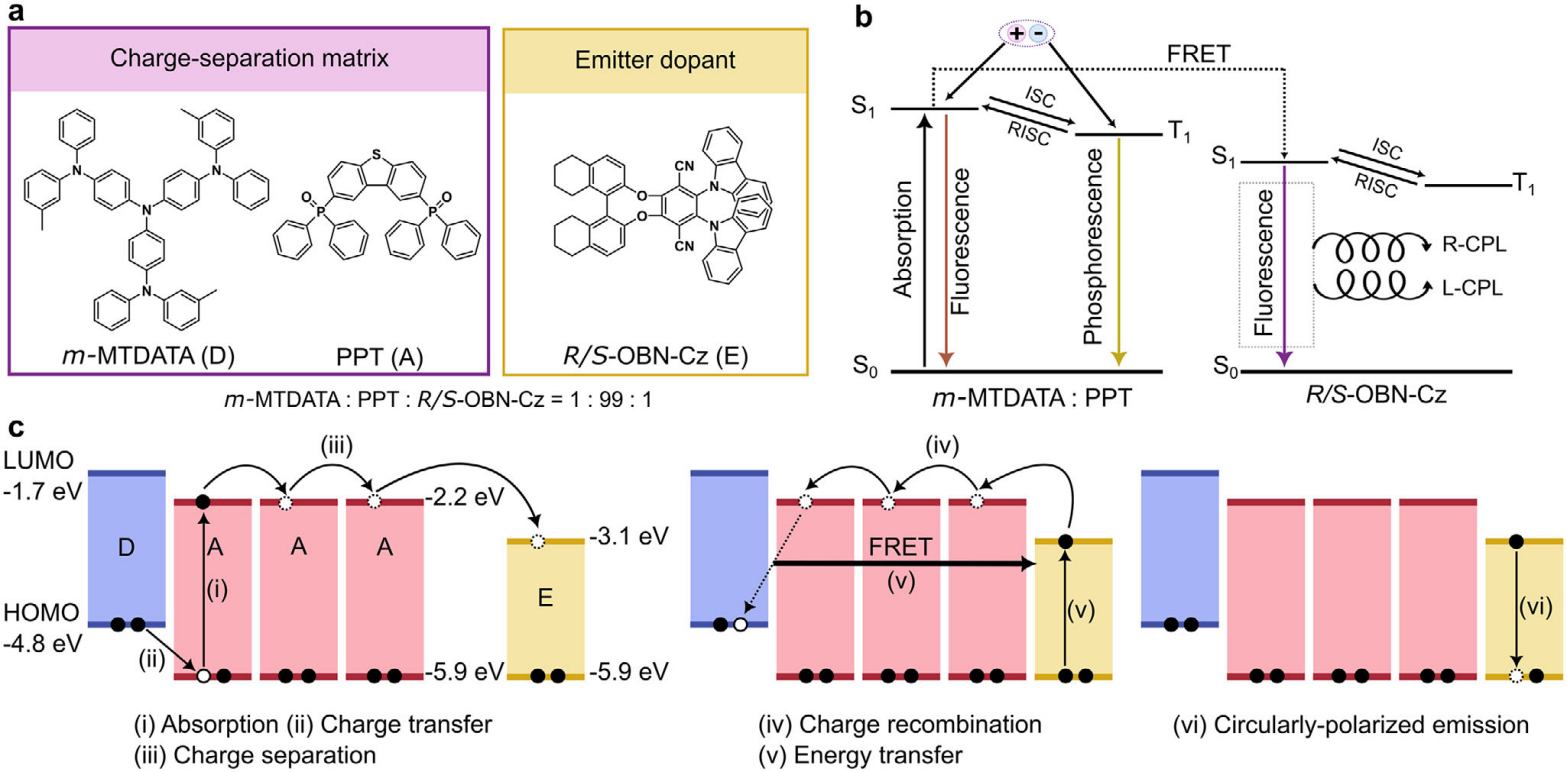
(a) Molecular structures of charge‐separation matrix (*m‐*MTDATA, PPT) and emitter dopant (*R/S*‐OBN‐Cz). (b) Energy diagram of charge‐separation matrix and the emitter dopant. (c) HOMO and LUMO energy levels of materials and CP‐LPL mechanism of three‐component FRET system.

In this work, we introduce long‐persistent and photostimulated luminescence with CPL characteristics in purely organic materials. To demonstrate CP‐LPL, we explore two design strategies: (i) three‐component Förster resonance energy transfer (FRET) system, in which a donor–acceptor matrix acts as a charge generator and transfers energy to a chiral emitter (Figure 1); and (ii) a two‐component upconversion system, where the locally excited (LE) state of the chiral emitter is restored upon charge recombination (Figure 3). In addition, three‐component FRET system shows CP‐PSL by NIR stimulated release of trapped charges and subsequent energy transfer to the chiral emitter (Figure 4). This study provides new proof of concept in chiroptical materials research rather than an immediately device‐ready technology, in which the need for optimization toward practical use will be further realized.

## 2 Results and Discussion

### 2.1 Photophysical, Chiroptical, and Electrochemical Properties of *R/S*‐OBN‐Cz

*R*‐ and *S*‐OBN‐Cz were chosen as chiral emitters for the CP‐LPL and CP‐PSL systems [35]. Both enantiomers exhibited a strong π–π* absorption band at 300–340 nm and a weaker intramolecular CT band centered around 420 nm (Figure S1). The emission maxima at 508 nm with photoluminescence quantum efficiency (PLQY) values ≈ 64%, mirror‐image circular dichroism (CD) and CPL spectra with |*g*_PL_| ≈ 2.9 × 10^−4^ were observed (Figure S2). The highest occupied molecular orbital (HOMO) and the lowest unoccupied molecular orbital (LUMO) energy levels of *R/S*‐OBN‐Cz were estimated to be −5.9 and −3.1 eV respectively, using differential pulse voltammetry (DPV) (Figure S3). On the basis of these values, *m*‐MTDATA/PPT was selected as charge‐separation matrix for the three‐component FRET system, while *m*CBP was chosen as the donor matrix for the two‐component upconversion system [31, 36].

### 2.2 CP‐LPL of Three‐Component FRET System

Amorphous melt‐cast films (∼50 µm thick) were prepared with a composition of *m‐*MTDATA:PPT:*R/S*‐OBN‐Cz = 1:99:1 molar ratio (Figure 1a). X‐ray diffraction spectra showed only broad halos, confirming the absence of diffraction from crystalline structure (Figure S4a). In this system, the *m‐*MTDATA/PPT matrix stored long‐lived CS states, while *R/S*‐OBN‐Cz acted as an electron trap and a final emitter, without interfering with charge separation (Figure 1c).

The *m*‐MTDATA/PPT is a known OLPL system, in which the radical cations of *m‐*MTDATA (*m‐*MTDATA^•^^+^) and the radical anions of PPT (PPT^•^^−^) are formed upon photoexcitation and can persist for hours at room temperature [37]. The slow recombination of *m‐*MTDATA^•^^+^ and PPT^•^^−^ results in singlet and triplet CT excited states with broad intermolecular CT emission peaks around 520 nm, which overlap with the intramolecular CT absorption band of *R/S*‐OBN‐Cz (Figure 1b,c; Figure S5a). This spectral overlap between CT emission and *R/S*‐OBN‐Cz absorption induces FRET, effectively quenching the CT emission across all time domains and resulting emission originating from *R/S*‐OBN‐Cz. The steady‐state PLQY of *m‐*MTDATA:PPT:*R/S*‐OBN‐Cz was 26%, which is lower than that measured in solution. This decrease is likely attributable to a competing charge‐separation pathway. In the three‐component system, the CT excited state is formed between *m*‐MTDATA and PPT, and not between *m*‐MTDATA and *R/S*‐OBN‐Cz. The doping concentrations of *m*‐MTDATA and *R/S*‐OBN‐Cz are each 1% relative to PPT, so the average distance between these species is sufficiently large to suppress direct CT formation. In a two‐component film with *m*‐MTDATA:*R/S*‐OBN‐Cz = 1:1, a CT emission band is observed around 700 nm (Figure S5b). However, this CT emission is absent in the three‐component films, further confirming that direct CT between *m*‐MTDATA and *R/S*‐OBN‐Cz does not occur under the present conditions.

**FIGURE 2:**
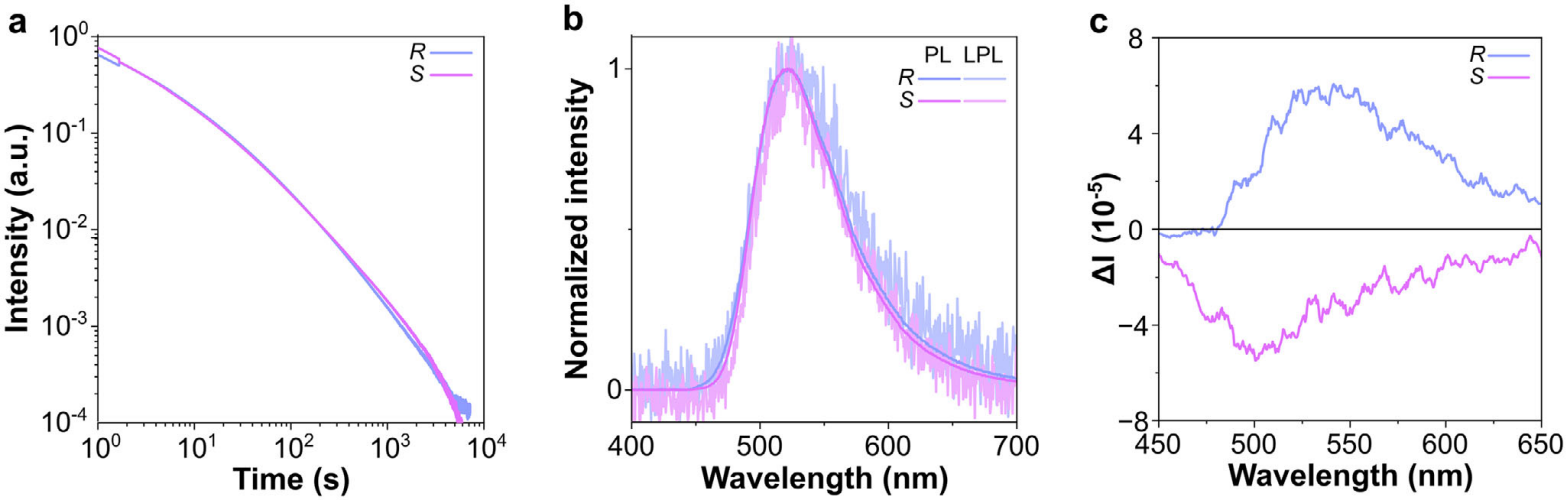
(a) Emission decay profiles of *m‐*MTDATA:PPT:*R/S*‐OBN‐Cz films on a log‐log plot. (b) Steady‐state PL and LPL spectra of *m‐*MTDATA:PPT:*R/S*‐OBN‐Cz after photoexcitation (excitation condition: excitation wavelength 365 nm, excitation power 1 mW, excitation time 60 s, 300 K, under nitrogen) (c) CPL spectra of *m‐*MTDATA:PPT:*R/S*‐OBN‐Cz.

Both films containing different enantiomers exhibited power‐law LPL decays extending beyond 2 h (Figure 2a; Figure S6a), providing evidence for efficient charge storage and recombination emission [38]. Steady‐state PL and LPL spectra were identical to those of neat *R/S*‐OBN‐Cz films, whereas the charge separation matrix showed green intermolecular CT emission (Figure 2b; Figure S5c). This confirms that nearly all stored energy is transferred to the chiral dopant via FRET. The films of *R/S*‐OBN‐Cz enantiomers displayed mirror‐image steady‐state CPL with |*g*_PL_| ≈ 2.8 × 10^−4^ (Figure 2c; Figure S7a). Because the |*g*_PL_| is extremely low, we were unfortunately unable to directly observe the CPL signal from the delayed component. However, the steady‐state and delayed PL spectra are in perfect agreement, in which the final emissive species are *R‐* and *S*‐OBN‐Cz, and the steady‐state |*g*_PL_| of the film matches that of *R/S*‐OBN‐Cz itself. Therefore, the LPL emission is also considered to be CPL in the same manner [30, 39].

### 2.3 CP‐LPL of Two‐Component Upconversion System

**FIGURE 3:**
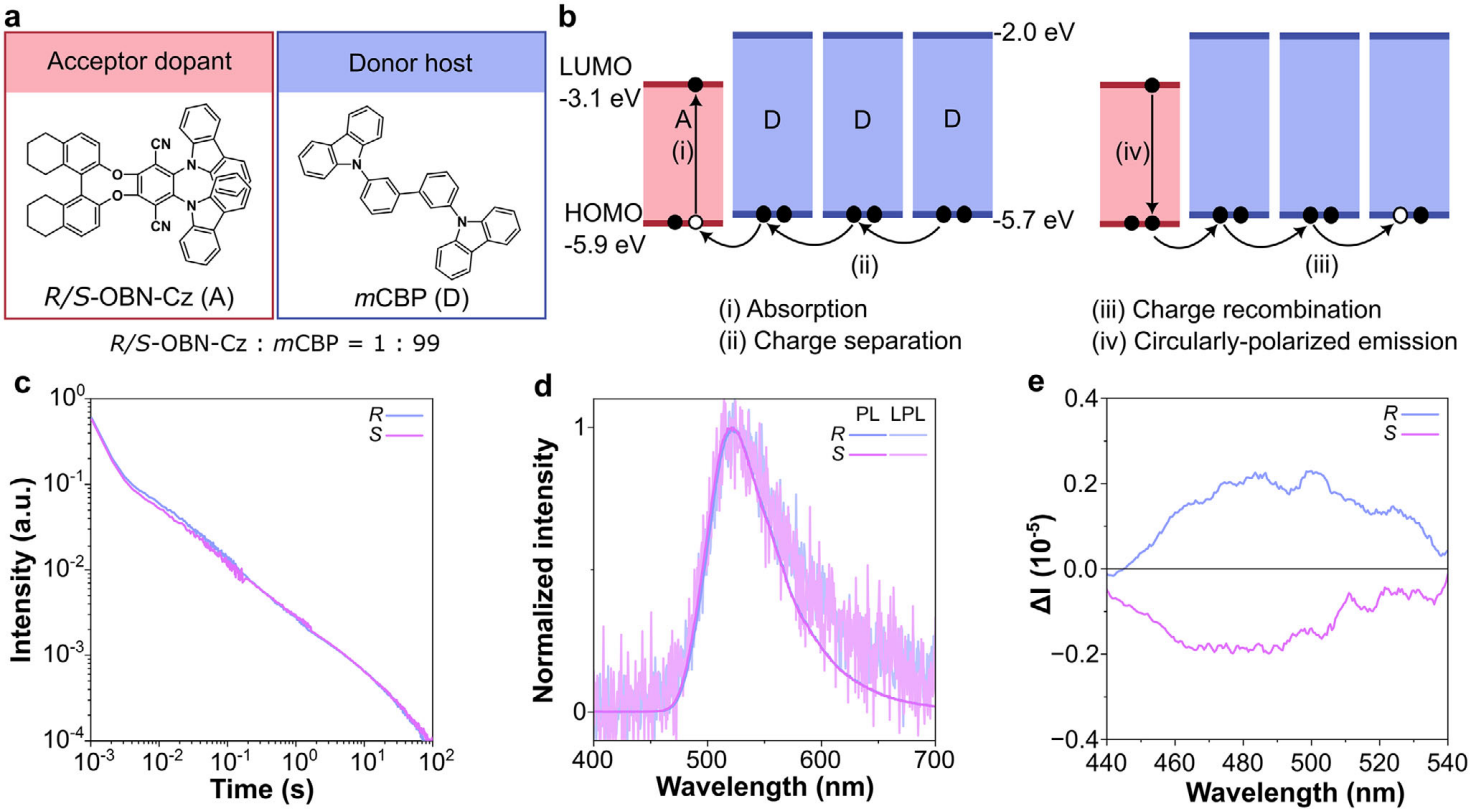
(a) Molecular structure of acceptor dopant (*R/S*‐OBN‐Cz) and donor host (*m*CBP). (b) HOMO and LUMO energy levels of materials and the CP‐LPL mechanism of two‐component upconversion system. (c) Emission decay profiles of *R/S*‐OBN‐Cz:*m*CBP films on a log‐log plot. (d) Steady‐state PL and LPL spectra of *R/S*‐OBN‐Cz:*m*CBP after photoexcitation (excitation condition: excitation wavelength 365 nm, excitation power 1 mW, excitation time 60 s, 300 K, under nitrogen). (e) CPL spectra of *R/S*‐OBN‐Cz:*m*CBP.

To produce CP‐LPL through the upconversion system, we designed two‐component amorphous systems with closely matched HOMO energy levels of the chiral acceptor dopants (*R*‐ or *S*‐OBN‐Cz, 1 mol%) and the donor host (*m*CBP, 99 mol%). The HOMO energy offset of 0.2 eV enabled the upconversion of the excited state from intermolecular CT to LE of the chiral dopants (Figure 3a,b; Figure S4b) [21].

The LPL decay profiles of two films containing different enantiomers (Figure 3c; Figure S6b) exhibited identical power‐law behavior, with durations of around 100 s. The steady‐state PL and LPL spectra exhibited a consistent single peak emission at 522 nm, corresponding to the *R/S*‐OBN‐Cz without intermolecular CT emission (Figure 3d; Figure S5d). In this hole diffusion type OLPL system [36], the hole is generated upon excitation in HOMO of *R/S*‐OBN‐Cz, and is filled with an electron from HOMO of *m*CBP (Figure 3b). The steady‐state PLQY of *R/S*‐OBN‐Cz:*m*CBP was 88%. Although the PLQY is high for the two‐component system, the short LPL duration of the two‐component system is attributed to the absence of well‐defined charge traps. In the three‐component system, the dopant *R/S*‐OBN‐Cz can also function as an electron trap, thereby stabilizing the CS state more effectively. In contrast, in the two‐component system, charges accumulate in the intrinsic traps of the host *m*CBP, leading to a reduced ability to maintain the CS state.

Transient photoluminescence (TRPL) measurements also confirmed the upconversion to the LE state of *R/S*‐OBN‐Cz (Figure S8). The TRPL decay profiles of both films featured two exponential decay behaviors: (i) prompt fluorescence (ns region) and (ii) TADF (µs region, Figure S8a). The TRPL spectra in different time scales (41 ns, 4.3 µs, 690 µs) showed identical emission, verifying that both prompt and TADF emissions originated from LE of *R/S*‐OBN‐Cz (Figure S8b).

The chiroptical properties of both films containing different enantiomers were studied using CPL spectroscopy (Figure 3e; Figure S7b), and the opposite signal profiles with the |*g*_PL_| ≈ 1.3 × 10^−4^ were obtained. These results confirm that when the HOMO levels of host and chiral dopant are closely aligned, CP‐LPL can be efficiently generated via LE emission, thus avoiding the weak inherent CT‐based CPL emission.

### 2.4 CP‐PSL of Three‐Component FRET System

**FIGURE 4:**
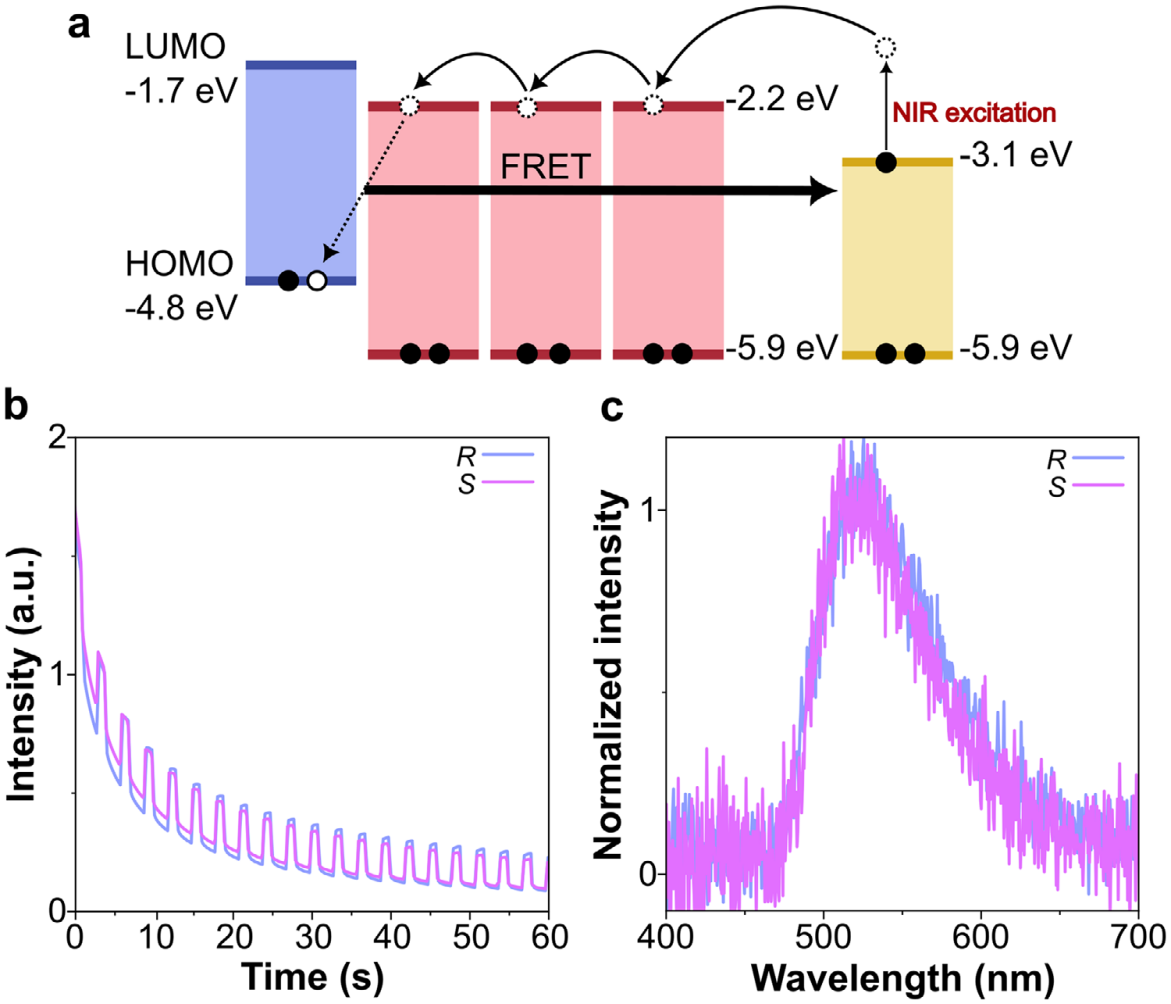
(a) CP‐PSL mechanism of three‐component FRET system. (b) The response of PSL intensity of *m‐*MTDATA:PPT:*R/S*‐OBN‐Cz (photoexcitation: excitation wavelength 365 nm, excitation power 20 mW, excitation time 60 s, 300 K and photostimulation: excitation wavelength 940 nm, excitation power 15 mW, 300K, ambient condition). (c) PSL spectra of *m‐*MTDATA:PPT:*R/S*‐OBN‐Cz.

After the UV‐excitation of the three‐component FRET system *m‐*MTDATA:PPT:*R/S*‐OBN‐Cz (1:99:1 molar ratio), radical anions of *R/S*‐OBN‐Cz (*R/S*‐OBN‐Cz^•^^−^) are formed, acting as traps. The absorption spectra of the *R/S*‐OBN‐Cz^•^^−^ feature the NIR band till 950 nm, and this wavelength can be used as a second stimulation. Therefore, by NIR irradiation, it is possible to stimulate the release of electrons from the trapped state (Figure 4a; Figure S9).

The two CP‐LPL films showed identical signal profiles during NIR stimulation (Figure 4b). Each profile reflects the trapped carriers released, promoting recombination and emission. Additionally, the PSL spectra of both *R*‐ and *S*‐form were identical, corresponding to the *R/S*‐OBN‐Cz emission in neat films (Figure 4c). This demonstrates that the trapped charges in the chiral dopant could be released upon NIR stimulation, regenerating circularly polarized emission on demand.

## 3 Conclusion

In summary, we have demonstrated CP‐LPL and CP‐PSL using the chiral emitter *R/S*‐OBN‐Cz in organic systems. Two design strategies were developed to achieve CP‐LPL: (i) FRET‐based approach – incorporating a CPL emitter into the donor/acceptor OLPL system, where energy is transferred from the charge‐separation matrix to the chiral emitter; (ii) upconversion‐based approach – making a two‐component OLPL system with adjusted frontier orbital energies, allowing upconversion of the intermolecular CT excited state to the LE state of the chiral component. Both approaches produced CP‐LPL with clear mirror‐image CPL signals, highlighting the versatility of organic systems. Additionally, in the three‐component FRET system, charges trapped in the chiral emitter could be released upon NIR stimulation, realizing the CP‐PSL.

These results establish a proof of concept for achieving CP‐LPL and CP‐PSL in purely organic systems. The observed |*g*_PL_| values are relatively small, as is typical for organic materials composed of light elements, where spin‐orbit coupling is intrinsically weak and the magnetic transition dipole moment is much smaller than the electric transition dipole moment [40, 41]. Nevertheless, higher |*g*_PL_| values have been reported for organic emitters by employing heavy‐atom‐containing luminophores (e.g., lanthanide complexes) and by introducing higher‐order (supramolecular) structures that enhance chiroptical responses [42, 43]. Accordingly, we expect that further optimization of the emitter and its chiral environment will lead to more efficient CP‐LPL and CP‐PSL. We anticipate that the present framework will serve as a versatile basis for the future development of chiroptical persistent luminescent technologies.

## 4 Experimental Section

### 4.1 Material Characterizations

The 4,4',4''‐tris[(3‐methylphenyl)phenylamino]triphenylamine (*m‐*MTDATA) was purchased from Merck, 2,8‐bis(diphenyl‐phosphoryl)‐dibenzo[*b,d*]thiophene (PPT), 3,3′‐di(9*H*‐carbazol‐9‐yl)‐1,1′‐biphenyl (*m*CBP), and *R/S*‐2,3‐di(9*H*‐carbazol‐9‐yl)‐8,9,10,11,12,13,14,15‐octahydrobenzo[*b*]dinaphtho[2,1‐*e*:1',2'‐*g*][1,4]dioxocine‐1,4‐dicarbonitrile (*R/S*‐OBN‐Cz) were prepared, according to the previous reports [31, 35, 36, 44]. All compounds were purified by train sublimation. ^1^H‐ and ^13^C‐NMR spectra were recorded on a Bruker AVANCE III 500 MHz with CDCl_3_ as a solvent. High‐resolution mass spectra (HRMS) were recorded by matrix‐assisted laser desorption/ionization time‐of‐flight spectrometry (MALDI‐TOF) using a Bruker MALDI ulthrafleXtreme. UV–Vis absorption and fluorescence emission spectra were recorded on a fluorescence/absorbance spectrometer Horiba Duetta. Absolute photoluminescence quantum yields (PLQY) were measured on an integrating sphere with a photoluminescence measurement unit (Quantaurus‐QY, C11347‐01, Hamamatsu Photonics). The redox potentials of *R/S*‐OBN‐Cz were obtained by differential pulse voltammetry (DPV) measurements (Figure S3) using BAS, ALS610E equipped with three electrodes (a glassy carbon working electrode, a platinum fiber counter electrode, Ag/Ag^+^ reference electrode) in dried tetrahydrofuran and *N*,*N*‐dimethylformamide using 0.1 m tetrabutylammonium hexafluorophosphate (TBAPF_6_) as a supporting electrolyte. Redox potentials were referenced against ferrocene/ferrocenium (Fc/Fc^+^) [45]. The radical anions of *R/S*‐OBN‐Cz were generated by electrical reduction on a honeycomb spectroelectrochemical electrode (PINE, honeycomb spectroelectrochemistry cell kit) in a DMF solution that contained 0.1 m TBAPF_6_ using an electrochemical analyzer (PINE, WaveNow). The circular dichroism (CD) spectra were measured on a JASCO J‐820 spectrometer. The CPL spectra were measured using a JASCO CPL‐300 (light source: 150 W xenon lamp) at room temperature. The excitation wavelength was 365 nm, with a scan rate of 50 nm/min and an accumulation of 8 times to improve signal‐to‐noise ratio and suppress random noise contributions. Artifacts in the spectra were confirmed by inverting the samples and changing their angle (±45 degrees), which did not affect the measured dissymmetry factors. Time‐resolved decay profiles were measured with iCCD (Princeton Instruments PI‐MAX 4 1024×1024). To measure transient PL, the sample was excited by a Yb:KGW femtosecond laser (PHAROS, Light Conversion) with the optical parametric amplifier (ORPHEUS, Light Conversion). The 165 fs width pulse was set to 1 kHz frequency and 350 nm wavelength.

### 4.2 Sample Fabrication

The three‐component films, *m‐*MTDATA:PPT:*R/S*‐OBN‐Cz (1:99:1 molar ratio), and two‐component films, *R/S*‐OBN‐Cz:*m*CBP (1:99 molar ratio) were prepared by the method reported in the literature [37]. *R/S*‐OBN‐Cz were dissolved in dichloromethane, and mixed with *m*‐MTDATA:PPT powder or *m*CBP powder. The solvent was evaporated at 80°C under reduced pressure. After that, the mixture was grounded and placed onto glass substrates with a cavity (10 × 10 × 0.5 mm) and melted at 300°C. The fabrication of the films was conducted in a nitrogen‐filled glovebox.

### 4.3 LPL Measurements

The LPL decay profiles were collected by a silicon photomultiplier (MPPC module, C13366‐1350GA, Hamamatsu Photonics), while PL and LPL spectra were recorded by a multichannel photodetector (PMA‐12, C14631‐01, Hamamatsu Photonics). Samples were measured in a dark chamber in a glovebox at room temperature with excitation of a 365 nm LED with a bandpass filter of 365 ± 5 nm, 1 mW excitation power, and 60 s excitation duration. LED excitation power was measured by a power meter (S120VC, Thorlabs).

### 4.4 PSL Measurements

PSL decay profiles were obtained by a silicon photomultiplier (MPPC module, C13366‐1350GA, Hamamatsu Photonics), while PL and PSL spectra were recorded by a multichannel photodetector (PMA‐12, C14631‐01, Hamamatsu Photonics). Samples were placed in a dark chamber, then excited by a 365 nm LED (20 mW) from one side of the chamber, followed by photostimulation with a 940 nm LED (15 mW) from the other side. LED power output was measured with a power meter (S120VC, Thorlabs).

## Author Contributions

R.M. and R.K. conceived the idea and experiment. R.M., R.Y. and S.L.V.N.Y. synthesized and characterized the compounds. R.M., R.Y. and D.K. conducted the LPL and PSL measurements. K.T., H.S. and T.K. performed CPL measurements. R.M., D.K. and K.K. measured and analyzed TRPL. R.M. and R.K. wrote the manuscript. R.K. supervised the project. All authors discussed the results and commented on the manuscript.

## Conflicts of Interest

The authors declare no conflict of interest.

## Supporting information




**Supporting File**: advs73632‐sup‐0001‐SuppMat.pdf.

## Data Availability

The data that support the findings of this study are available from the corresponding author upon reasonable request.
