# Supplementary material for: Circularly Polarized Long‐Persistent and Photostimulated Luminescence Enabled through Förster Resonance Energy Transfer and Upconversion Strategies
Source: Adv Sci (Weinh). 2026 Jan 12;13(14):e23415. doi: 10.1002/advs.202523415 (PMC12970155; doi:10.1002/advs.202523415)
Supplement: Supplementary file 1 — Supporting File: advs73632‐sup‐0001‐SuppMat.pdf. [file ADVS-13-e23415-s001.pdf]

Supporting Information

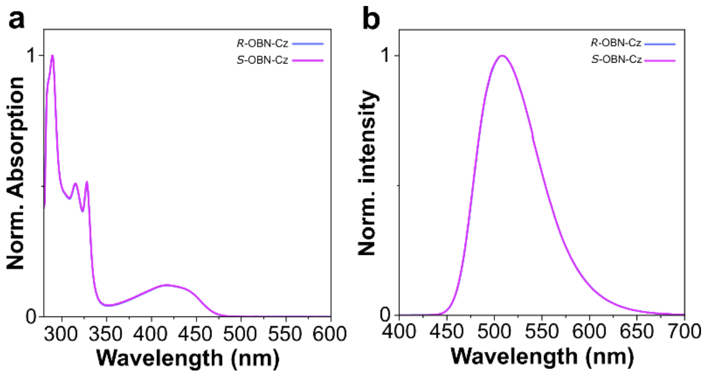

Figure S1. Normalized UV-Vis absorption (a) and emission (b) spectra of *R/S*-OBN-Cz in toluene solution.

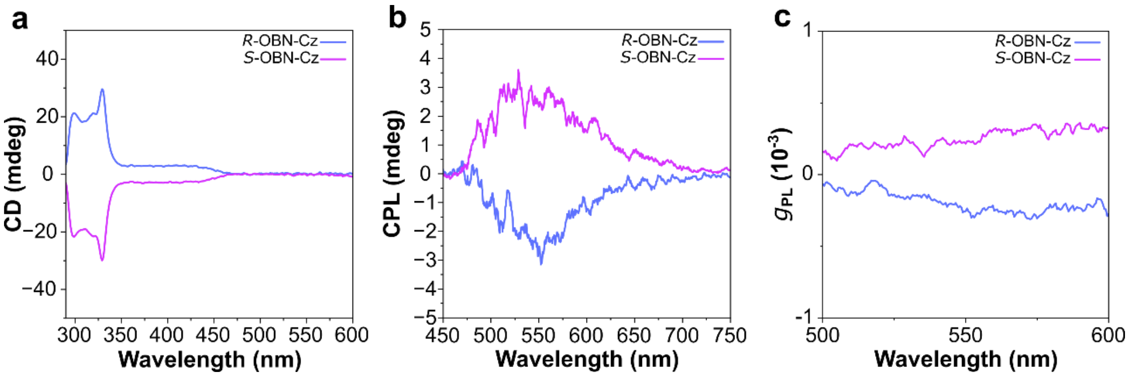

Figure S2. CD (a) and CPL (b) spectra and g_{PL} versus wavelength plots (c) of *R/S*-OBN-Cz in toluene solution.

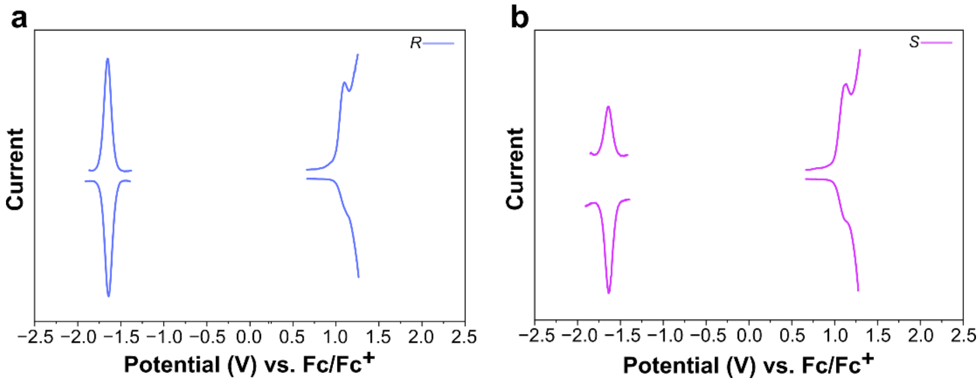

Figure S3. Differential pulse voltammogram of (a) *R*-OBN-Cz and (b) *S*-OBN-Cz.

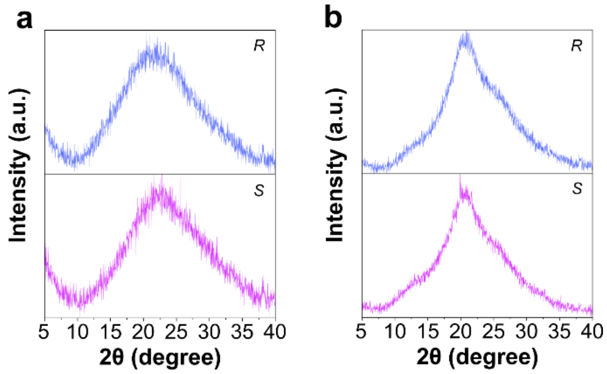

Figure S4. XRD patterns of (a) *m*-MTDATA:PPT:*R/S*-OBN-Cz and (b) *R/S*-OBN-Cz:*m*CBP.

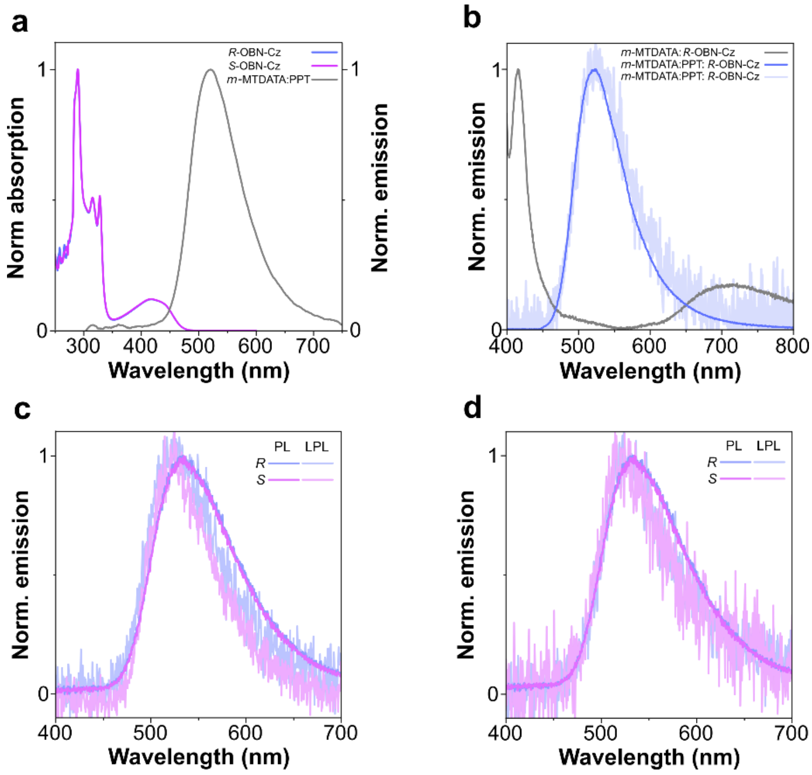

Figure S5. (a) UV-Vis absorption spectra of *R/S*-OBN-Cz and PL emission spectrum of *m*-MTDATA:PPT, (b) PL emission spectra of *R*-OBN-Cz:*m*-MTDATA (1:1) and PL/LPL emission spectra of *m*-MTDATA:PPT:*R/S*-OBN-Cz (1:99:1), (c) PL spectra of *R/S*-OBN-Cz (neat films) and LPL spectra of three-component films (*m*-MTDATA:PPT:*R/S*-OBN-Cz), and (d) PL spectra of *R/S*-OBN-Cz (neat films) and LPL spectra of two-component films (*R/S*-OBN-Cz:*m*CBP).

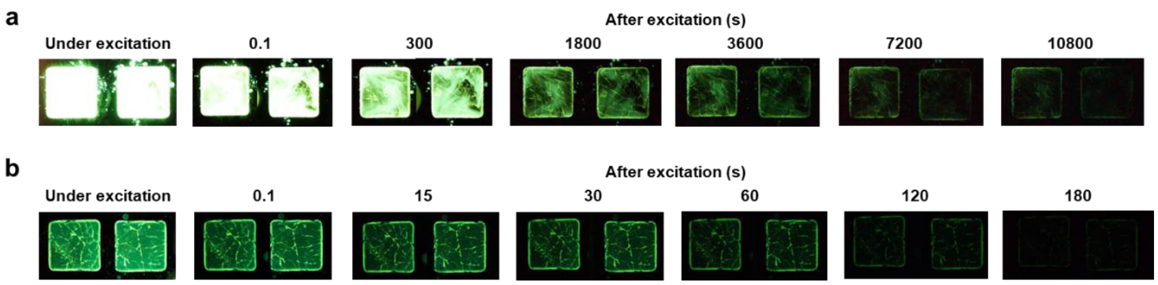

Figure S6. Photographs of (a) three-component FRET-based films (*m*-MTDATA:PPT:*R/S*-OBNCz) and (b) two-component films (*m*CBP:*R/S*-OBNCz) under UV excitation and after excitation; left-side for *R*- and right-side for *S*-forms.

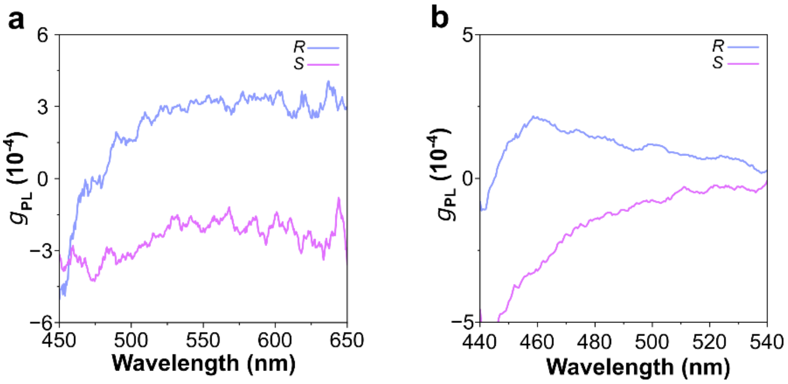

Figure S7. g_{PL} versus wavelength plots for (a) *m*-MTDATA:PPT:*R/S*-OBN-Cz and (b) *R/S*-OBN-Cz:*m*CBP films.

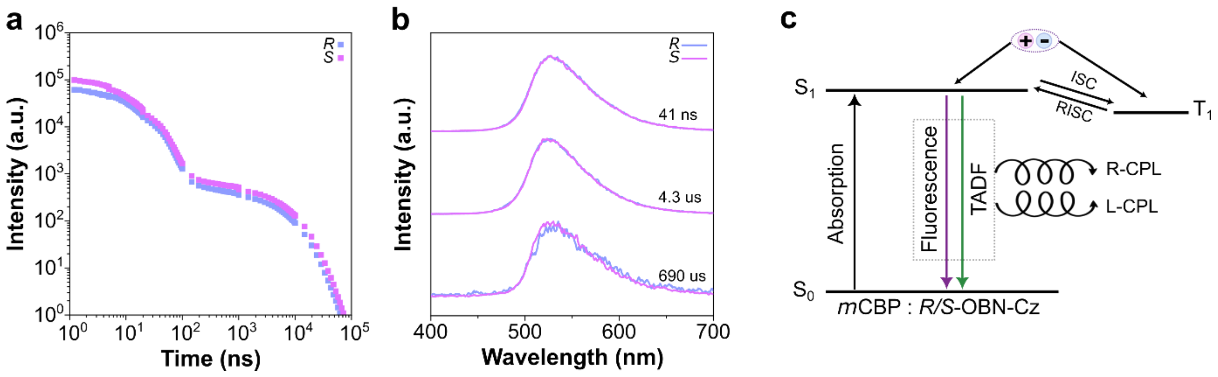

Figure S8. TRPL decays (a) and emission spectra (b) of two-component films (*R/S*-OBN-Cz:*m*CBP) and (c) Energy level diagram of *R/S*-OBN-Cz:*m*CBP system.

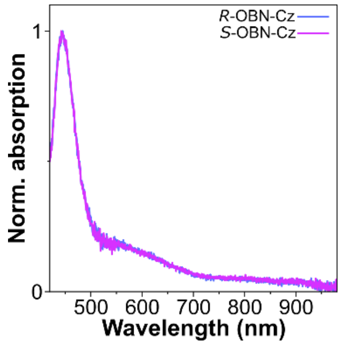

Figure S9. Normalized absorption spectra of *R/S*-OBN-Cz under electrochemical reduction in DMF solution.

Synthesis of *R/S*-OBN-F

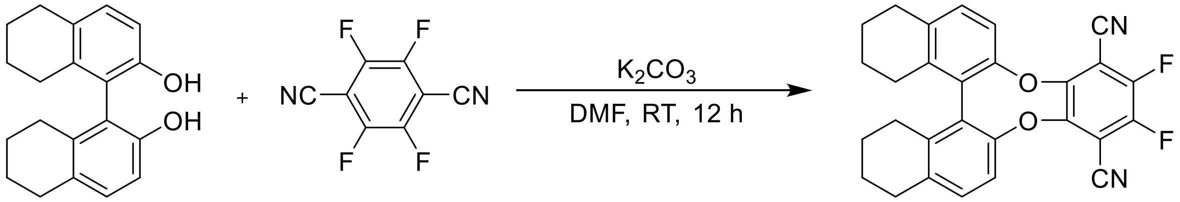

A mixture of *R/S*-5,5',6,6',7,7',8,8'-octahydro-1,1'-2-naphthol (*R/S*-OBN) (1.7 mmol), tetrafluoroterephthalonitrile (1.7 mmol), potassium carbonate (4.2 mmol) was dissolved and stirred in dry DMF (20 mL) under nitrogen atmosphere for 12 h at room temperature. The reaction mixture was extracted with ethyl acetate/water (3 times), and dried over Na_2SO_4, and concentrated under reduced pressure. The crude product was purified by flash column chromatography (ethyl acetate:hexane, 1:10) to give *R/S*-2,3-difluoro-8,9,10,11,12,13,14,15-octahydrobenzo[*b*]dinaphtho[2,1-*e*:1',2'-*g*][1,4] dioxocine-1,4-dicarbonitrile (*R/S*-OBN-F) as a white solid (85% yield). ^{1}H NMR (500 MHz, Chloroform-d) δ 7.15 (d, J = 8.3 Hz, 1H), 7.03 (d, J = 8.2 Hz, 1H), 2.85 (qt, J = 16.6, 6.1 Hz, 2H), 2.64 (ddd, J = 16.8, 8.8, 4.8 Hz, 1H), 2.38 (dt, J = 17.0, 5.9 Hz, 1H), 1.90 – 1.75 (m, 3H), 1.68 (pd, J = 8.5, 7.2, 3.9 Hz, 1H). ^{13}C NMR (126 MHz, Chloroform-d) δ 148.85, 147.82, 147.80, 137.22, 136.67, 130.40, 128.62, 117.81, 108.78, 29.04, 27.28, 22.27, 22.15. HRMS MALDI-TOF (cald. for $C_{28}H_{20}F_2N_2O_2$, *m/z* 454.1493): found [M^+] 454.1465

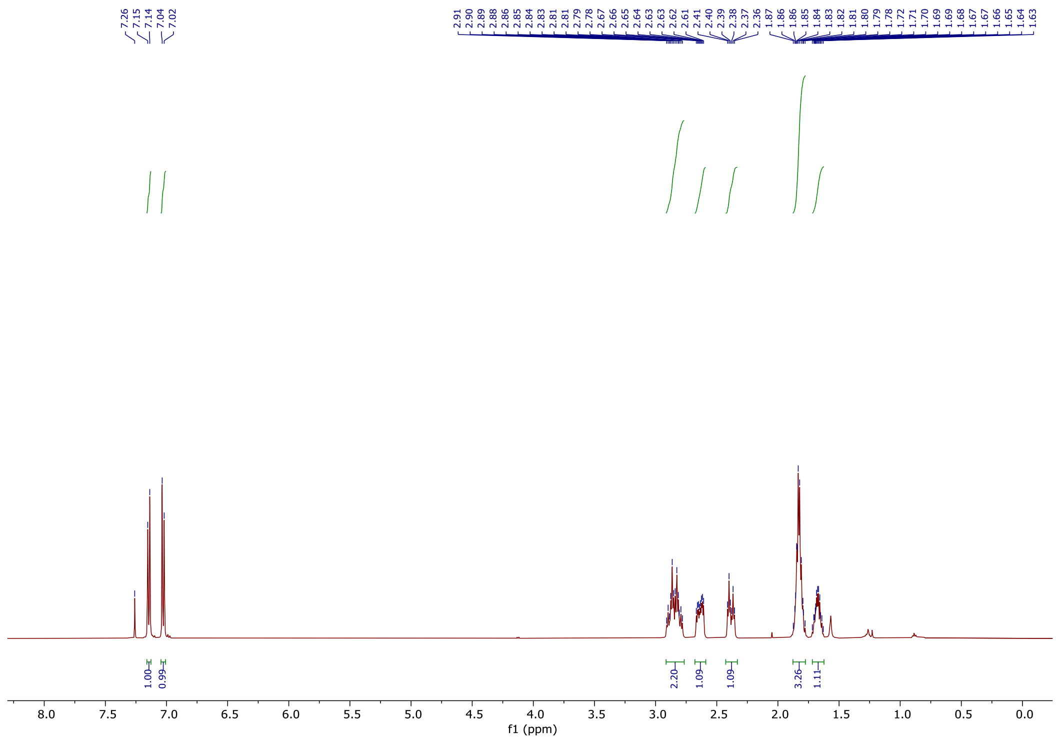

^{1}H-NMR spectrum of of *R/S*-OBN-F

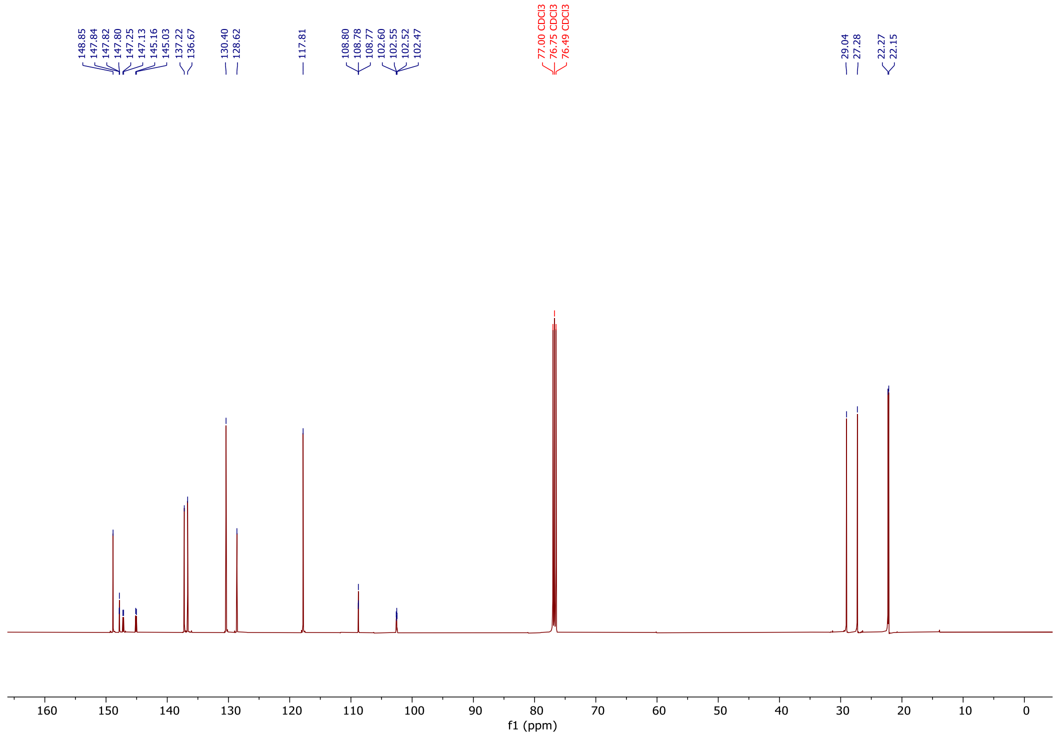

^{13}C-NMR spectrum of of *R/S*-OBN-F

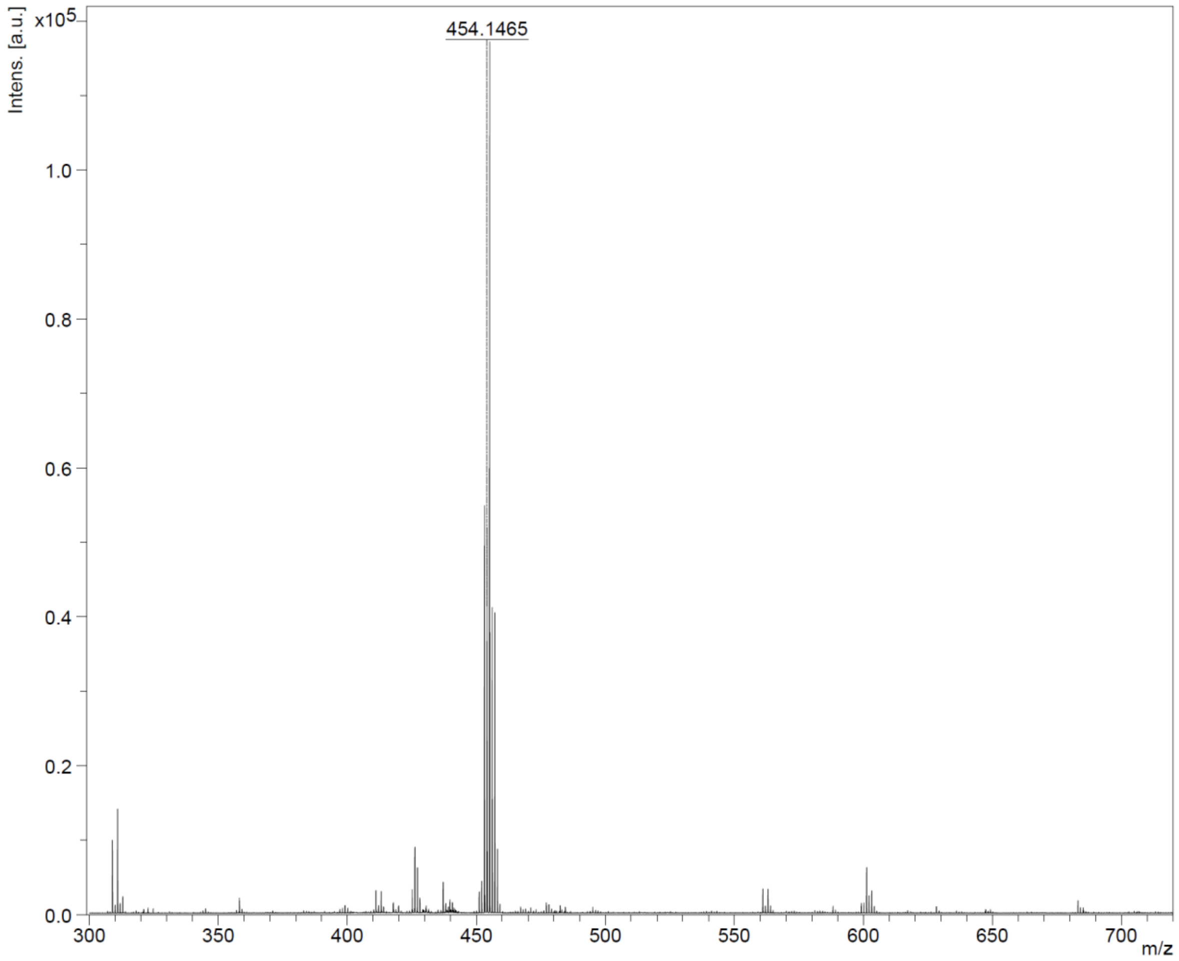

MASS spectrum of of *R/S*-OBN-F

Synthesis of *R/S*-OBN-Cz

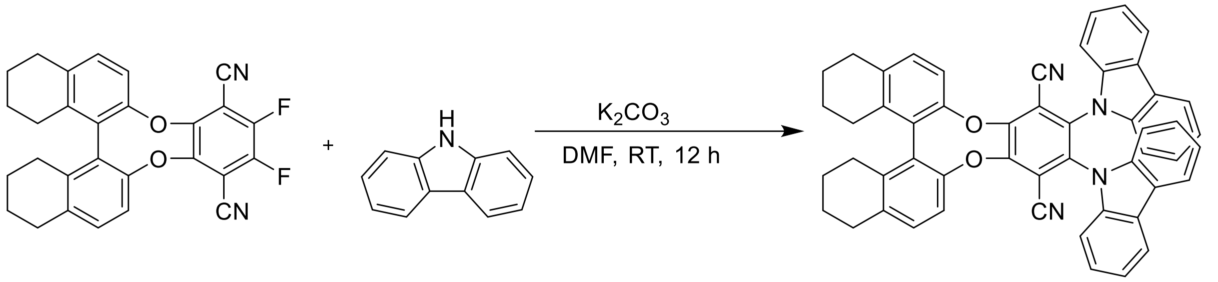

A mixture of *R/S*-OBN-F (0.66 mmol), carbazole (1.6 mmol), potassium carbonate (2.6 mmol) was dissolved and stirred in DMF (20 mL) under nitrogen atmosphere for 12 h at room temperature. The reaction mixture was extracted with ethyl acetate/water (3 times), and dried over Na_2SO_4, and concentrated under reduced pressure. The crude product was purified by flash column chromatography (ethyl acetate:hexane, 1:10) and subsequently sublimed to give *R/S*-2,3-di(9*H*-carbazol-9-yl)-8,9,10,11,12,13,14,15-octahydrobenzo[*b*]dinaphtho[2,1-*e*:1',2'-*g*][1,4]dioxocine-1,4-dicarbonitrile (*R/S*-OBN-Cz) as a yellow solid (75% yield). ^{1}H NMR (500 MHz, Chloroform-d) δ 7.73 (d, J = 7.6 Hz, 1H), 7.63 (d, J = 7.7 Hz, 1H), 7.26 – 7.19 (m, 3H), 7.17 – 7.11 (m, 2H), 6.94 (t, J = 7.5 Hz, 1H), 6.77 (td, J = 7.8, 7.1, 1.3 Hz, 1H), 6.66 (d, J = 8.2 Hz, 1H), 2.92 (qd, J = 16.5, 8.4 Hz, 2H), 2.72 (ddd, J = 16.8, 8.8, 4.7 Hz, 1H), 2.48 (dt, J = 17.0, 5.9 Hz, 1H), 1.89 (h, J = 6.9, 6.5 Hz, 3H), 1.74 (pd, J = 8.4, 7.1, 3.8 Hz, 1H). ^{13}C NMR (126 MHz, Chloroform-d) δ 152.34, 149.58, 139.18, 138.68, 137.87, 137.31, 134.76, 130.99, 129.32, 126.00, 125.32, 124.32, 124.13, 121.49, 121.21, 120.53, 120.11, 118.89, 114.57, 112.13, 109.95, 109.91, 29.68, 27.90, 22.87, 22.76. HRMS MALDI-TOF (cald. for $C_{52}H_{36}N_4O_2$, *m/z* 748.2838): found [M^+] 748.2834

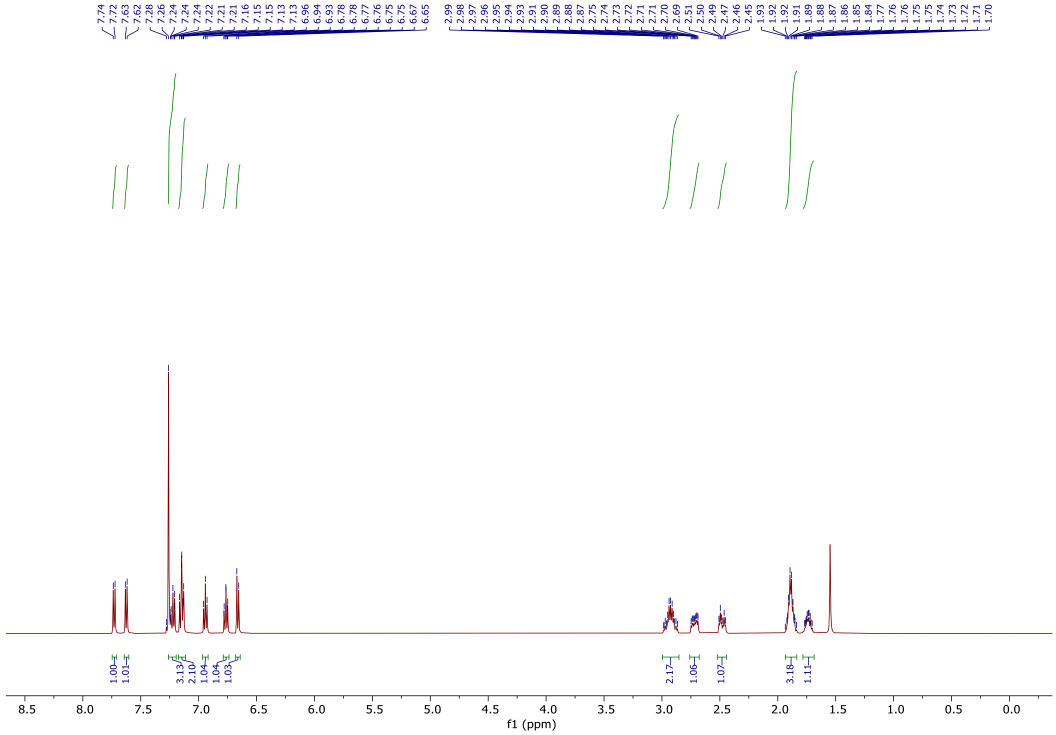

^{1}H-NMR spectrum of of *R/S*-OBN-Cz

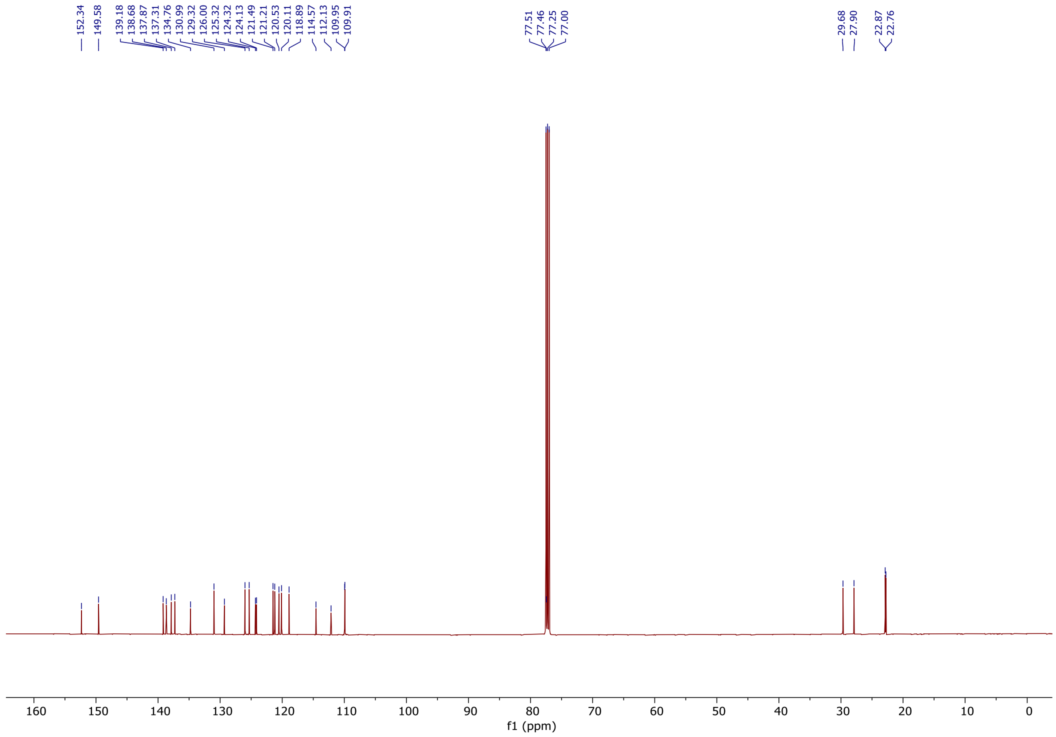

^{13}C-NMR spectrum of of *R/S*-OBN-Cz

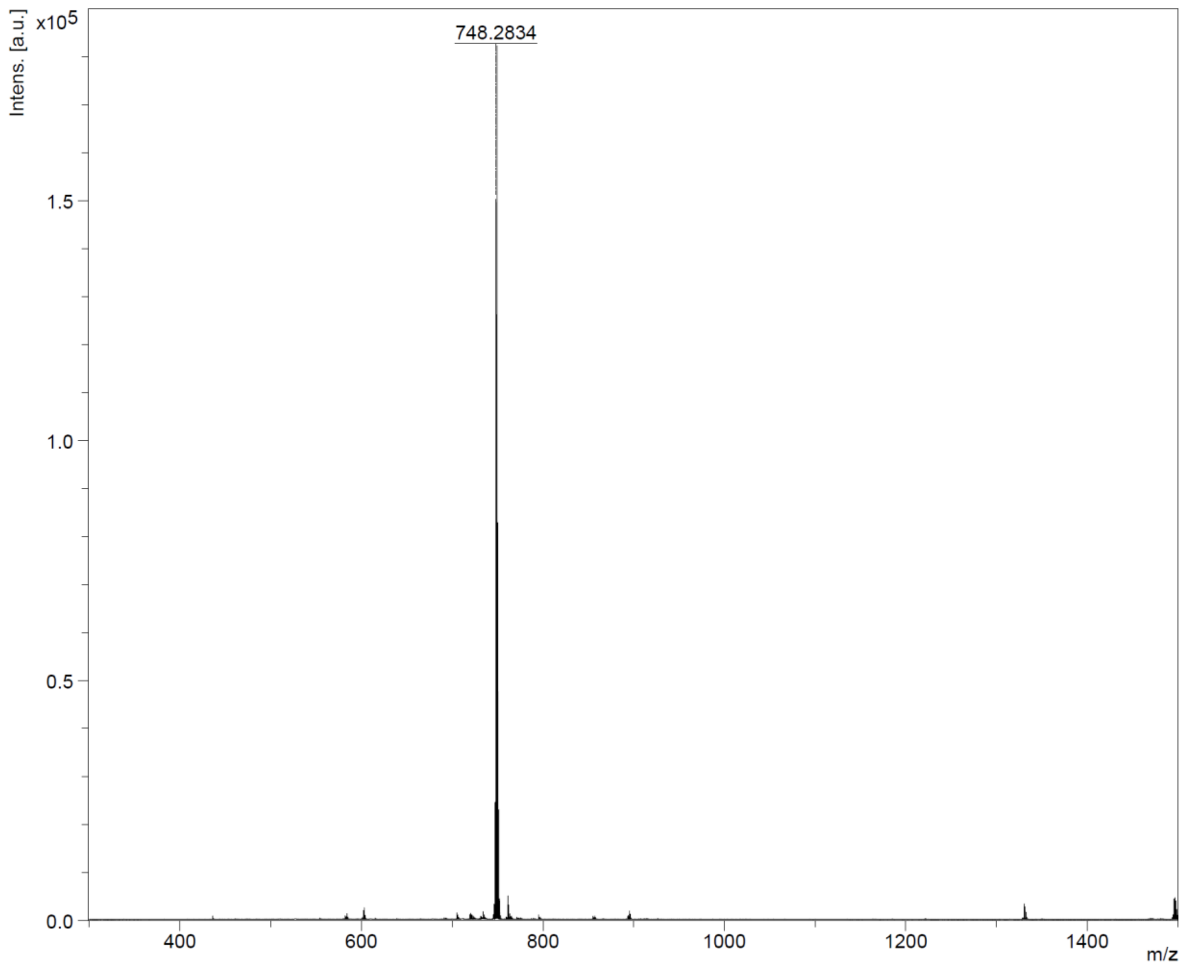

Mass spectrum of of *R/S*-OBN-Cz
